# Editorial: Modulation of pro-inflammatory signaling by interferons

**DOI:** 10.3389/fimmu.2025.1657424

**Published:** 2025-07-14

**Authors:** René Huber, Korbinian Brand, Kyung-Hyun Park-Min

**Affiliations:** ^1^ Institute of Clinical Chemistry/Central Laboratory, Hannover Medical School, Hannover, Germany; ^2^ Arthritis and Tissue Degeneration Program, David Z. Rosensweig Genomics Research Center, Hospital for Special Surgery, New York, NY, United States

**Keywords:** interferons, cytokines, mutual regulation, interferon-stimulated genes, inflammation, autoimmunity, interferon-associated diseases

Interferons (IFNs) are crucial regulators of the immune response that mediate both pro- and anti-inflammatory effects (1, 2). To date, the human IFN family comprises type I (IFN-I: IFN-α, -β, -ϵ, -κ, and -ω), II (IFN-γ), and III IFNs (IFN–λ). Following binding to their receptors, they activate intracellular signaling, predominantly the Jak–STAT pathway, and induce the expression of a broad range of interferon-stimulated genes (3). However, due to the variety of potentially activated cascades, shared target promoter elements, and the specific characteristics of interferon-responsive cells (among other things), different (types of) interferons may have distinct as well as overlapping functions. While their canonical function is the mediation of anti-viral activity and the defense against microbial infections, IFNs were further proven to be involved in anti-tumor immunity, autoimmunity, cell development, tissue protection, homeostasis, and metabolism (1, 4).

Under both physiological and pathophysiological conditions, however, interferons do not act on target cells alone but are part of a complex cocktail of cytokines, chemokines, growth factors, and other mediators, whose interaction defines the reaction of the cells to the specific stimulatory situation (5). Alterations in the proper orchestration of this immunomodulatory network may result in the dysregulations of the respective response, thus enabling infectious, inflammatory, or malignant diseases. Li et al., for instance, examine this aspect in the field of osteoimmunology, an interdisciplinary area studying the crosstalk between the immune system and the skeletal system. In their review, they focus on the intricate and in part contradictory impact of IFN-γ on bone remodeling during osteoporosis, a systemic disease characterized by low bone mass and an increased risk of fracture (6). The authors detail the multifaceted contribution of IFN–γ to osteoblast differentiation and the limitation of osteoclast formation but also consider its capacity to indirectly increase the bone-resorbing activity of mature osteoclasts. Thus, IFN–γ exhibits both osteoprotective and osteodestructive properties. In the case of osteoporosis, however, IFN-γ appears to account for a net loss of bone mass, although its effects may be complex and disease-stage-specific.

The contribution of type I IFNs to malignant diseases is taken up in the review by Meyer et al. Their article sheds light on the involvement of IFN–I signaling in myeloid anti-tumor immunity in the tumor microenvironment (TME). The authors describe how the activation of IFN-I-dependent signaling results in the reduced differentiation of monocytes to tumor-associated macrophages. Moreover, M2 macrophage polarization in the TME is shifted by IFN-I towards a pro-inflammatory M1-like phenotype with distinct anti-tumor activities, including enhanced phagocytosis, cytotoxicity, and T-cell recruitment. The authors conclude with the observation that activation of IFN–I responses in TME-macrophages represents a promising anti-tumorigenic approach. *Vice versa*, in the review by Lai et al., the therapeutic potential of targeting IFN-I and associated signaling is assessed in the context of systemic lupus erythematosus (SLE) and lupus nephritis (LN). Here, the diverse factors suggesting an involvement of IFN-I in the pathogenesis of SLE and LN are summarized, though the precise contribution of IFN-I to development and progression of these diseases is still under debate. By characterizing IFN–I as a central player in the complex molecular and cellular network leading to pro-inflammatory, -destructive, and -fibrotic events, the authors reveal the detrimental role of IFN-I in the kidney. In consequence, approaches targeting IFN-I and/or IFN-I-associated signaling represent potential treatment options. Current clinical trials, however, are not free of ambiguous or unexpected results, which makes generalized application difficult.

As illustrated, IFNs are of decisive importance in the development of (patho–)physiological states associated with inflammation, but their impact cannot be understood without knowledge of the events regulating their expression (1) or their interactions with other mediators (5). Therefore, Yu et al. have a closer look at interferon regulatory factor (IRF)5, a transcription factor that—due to the functional differentiation from its relatives—contributes to the expression of both IFN-I and pro-inflammatory cytokines/chemokines. The authors characterize IRF5 as a potent player in antiviral immunity but also as a driver of inflammatory and autoimmune diseases such as inflammatory bowel disease, SLE, or rheumatoid arthritis. Interestingly, IRF5 appears to worsen inflammation-associated malignancies, while it may adopt the role of a tumor suppressor in other forms of cancer, depending on the type of affected cells and tissues. Due to its involvement in these and other diseases, IRF5 can be regarded as a therapeutic target, and Yu et al. discuss various strategies to modulate expression, activation, function, and localization of IRF5. A reliable clinical application, though, is still pending.

Finally, two original research articles are dedicated to the role of single nucleotide polymorphisms (SNPs) in infectious diseases involving the modulation of interferons. Variations in genes coding for IFNs, IFN receptors, or IFN-inducing receptors, including pattern recognition receptors, are associated with the susceptibility for various diseases (7). In accordance with this, Santana et al. demonstrate the influence of toll-like receptor (TLR)7 SNPs rs179008 (A/T; amino acid exchange Gln11Leu) and rs3853839 (C/G; located in the 3’ untranslated region) on IFN–α and TLR7 levels in patients with human T–lymphotropic virus 1 infection. Though TLR7 genotypes had no influence on TNF expression or the symptoms of the infection, carriers of the rs179008 major allele exhibited increased IFN-α amounts, while the minor allele of rs3853839 was associated with higher TLR7 levels. Thus, the rs179008 major and the rs3853839 minor allele may have protective effects by supporting antiviral defense. Finally, Kaminiów et al. investigate the association of two SNPs located in introns of the IFN-γ gene, rs2430561 (T/A) and rs1861494 (A/G), with the serological response in early treated syphilis patients. In both cases, homozygous carriers of the major alleles showed higher IFN-γ levels in combination with a serological cure. In contrast, the homozygous minor genotype was associated with low IFN-γ levels and a (persisting) serofast state, an observation suggesting a predisposition for the development of serofast syphilis.

In summary, this Research Topic adds a few more mosaic tiles to a complex and still expanding topic and thus complements the picture we have of IFNs, their properties, and their interrelationship with other factors. The manuscripts included thus enhance our understanding of the molecular mechanisms underlying the diverse effects of IFNs under multiple conditions.
